# Metabolic Engineering of *Komagataella phaffii* and Process Optimization for Biosynthesis of 1,2,4‐Butanetriol From Xylose

**DOI:** 10.1002/biot.70085

**Published:** 2025-07-25

**Authors:** Débora Trichez, Thályta Pacheco, Clara Vida G. C. Carneiro, Jessica C. Bergmann, João Ricardo M. de Almeida

**Affiliations:** ^1^ Microbial Genetics and Biotechnology Laboratory EMBRAPA Agroenergy Brasília Brazil; ^2^ Graduate Program of Microbial Biology Institute of Biology University of Brasília Brasília Brazil

**Keywords:** 1,2,4‐butanetriol, bioprocess engineering, *Komagataella phaffii*, xylose

## Abstract

1,2,4‐butanetriol (BTO) is a four‐carbon polyol used as a precursor for synthesizing pharmaceuticals, polymers, and energetic plasticizers. The present study demonstrates the microbial production of BTO from xylose by engineered *Komagataella phaffii* yeast strains for the first time. The pathway was established through the overexpression of the enzymes xylose dehydrogenase (XylB), xylonate dehydratase (XylD), and 2‐ketoacid decarboxylase (KDC). Two xylonate dehydratase genes, *xylD‐CC* from *Caulobacter crescentus* and *xylD‐HL* from *Halomonas lutea*, were evaluated in the constructions, both enabling BTO production. Furthermore, to improve BTO production, a central composite design analysis (CCD) was employed, identifying the best cultivation conditions to improve yeast performance. Under these optimized conditions, the engineered *K. phaffii* strain produced 1.3 g/L of BTO, achieving a 147% increase compared to the initial setup. Although further genetic engineering efforts are required to enhance BTO production, this study provides insights into potential improvement targets and highlights *K. phaffii* as a promising platform for the bio‐based synthesis of chemical compounds like BTO.

## Graphical Abstract and Lay Summary

The yeast *K. phaffii* was engineered to produce butanetriol from xylose employing a modified Damhs pathway. The optimization of oxygen availability, culture pH, and initial cell density resulted in a 147% increase in renewable butanetriol production in batch fermentation.

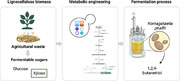

## 1 Introduction

Lignocellulosic biomass is an abundant, renewable energy and carbon source that can reduce or replace fossil‐based raw materials in the production of fuels and chemicals. Rich in carbohydrates, it is widely used as a feedstock in microbial fermentations to produce bio‐based compounds through more sustainable processes [1, 2].

Xylose, the second most abundant sugar present in the lignocellulose, is assimilated by microorganisms via different metabolic routes, including the oxidoreductive pathway, isomerase pathway (both linked with the pentose phosphate pathway—PPP), and non‐phosphorylating pentose oxidation pathway [3]. Furthermore, with the advancement of biology systems and synthetic biology techniques, new synthetic pathways have been implemented in microorganisms to achieve the production of chemicals that are not produced naturally by the cells, significantly increasing the range of products that can be obtained through microbial conversion. Indeed, the oxidative xylose pathway, with some additional modifications, has been explored into industrial microorganisms such as *Escherichia coli, Saccharomyces cerevisiae*, and *Komagataella phaffii*, leading to synthesis of several compounds, including xylonic acid [4, 5], ethylene glycol [6 ^–^ 8], glycolic acid [9, 10], 3,4‐dihydroxybutyrate [11], and 1,2,4‐butanetriol [12, 13, 14].

Among these products, 1,2,4‐butanetriol (BTO) has been attracting considerable attention. BTO is a four‐carbon polyol widely used as a building block for the synthesis of several chemicals for pharmaceutical and polymer industries as well as can be employed as a precursor of 1,2,4‐butanetriol trinitrate (BTTN), an energetic plasticizer applied in propellant and explosive formulations [13, 15]. BTO is usually manufactured through chemical synthesis using harsh reaction conditions and multiple steps, which results in a high‐cost production process, poor selectivity, and environmental pollution [13]. Considering these hurdles, the microbial synthesis of BTO presents a compelling alternative.

**FIGURE 1:**
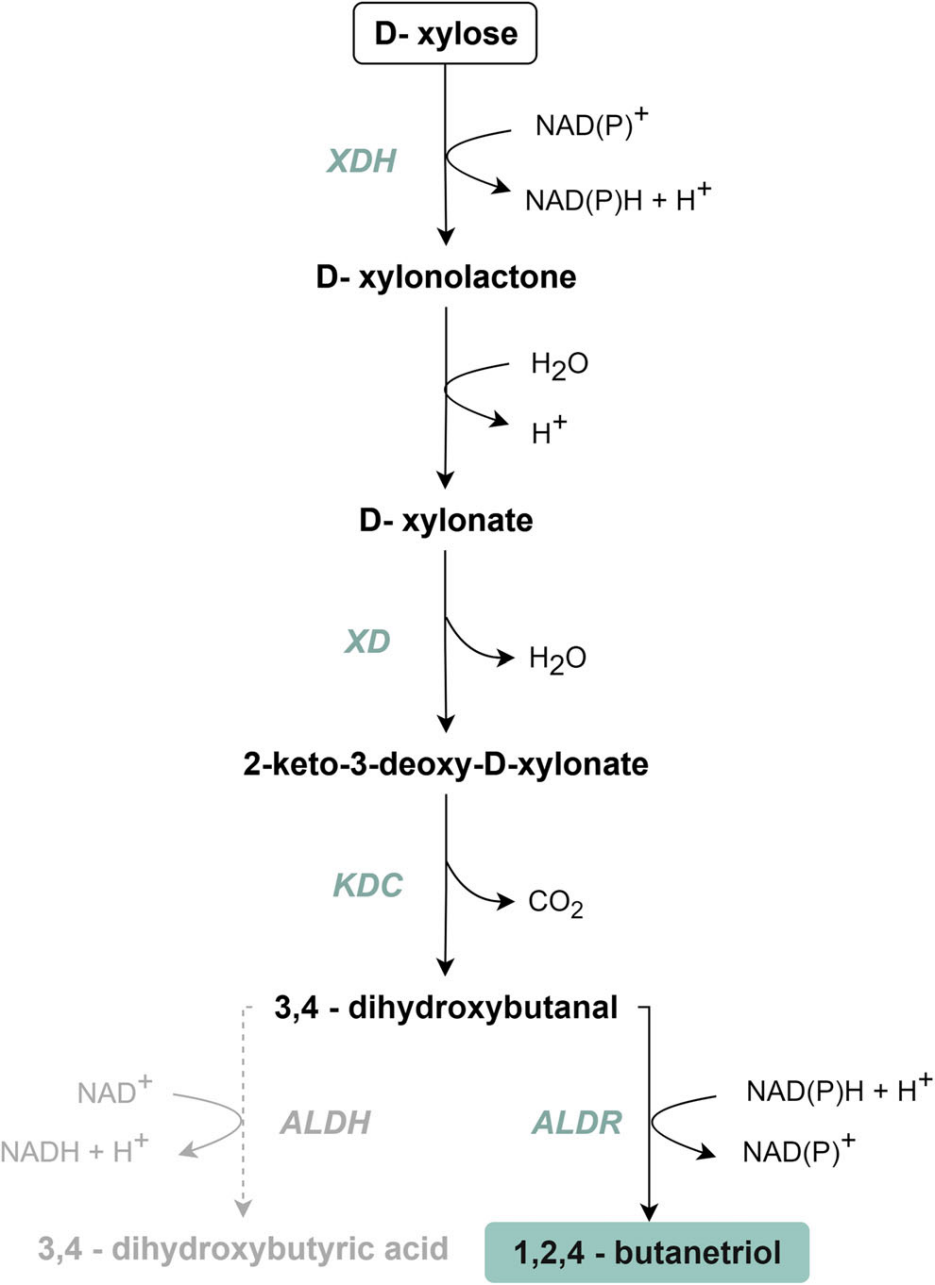
Metabolic pathway for 1,2,4‐butanetriol (BTO) production from xylose. Metabolite intermediates: xylonate (XA); 2‐keto‐3‐deoxy‐xylonate (KDX). Enzymes: XDH, xylose dehydrogenase; XLA, xylonolactonase; XD, xylonate dehydratase; KDC, 2‐keto acid decarboxylase; ALDR, aldehyde reductase; ALDH, aldehyde dehydrogenase.

The BTO production pathway from xylose consists basically of four steps (Figure 1). Initially, xylose is converted to xylonate by the enzyme xylose dehydrogenase (XDH), followed by a spontaneous reaction or action of a lactonase. The xylonate is dehydrated into 2‐keto‐3‐deoxy‐xylonate (KDX), a reaction catalyzed by the xylonate dehydratase (XD). Then, the intermediate KDX is decarboxylated by a 2‐keto acid decarboxylase (KDC) into 3,4‐dihydroxybutanal, which can be reduced to BTO by action of aldehyde reductase or alcohol dehydrogenase enzymes [3]. Production of BTO from xylose was first achieved using a sequential process combining *Pseudomonas fragi*, which provides the xylose oxidation to xylonate, followed by *E. coli* conversion of xylonate into BTO [14]. After that, other recombinant *E. coli* strains have been constructed for BTO production [13, 15], and only more recently, the yeasts *S. cerevisiae* and *Candida tropicalis* have also been engineered with this proposed pathway [12, 16, 17].

The yeast *K. phaffii*, another relevant industrial microorganism, has attracted increasing attention to be explored as a platform to produce chemical compounds, besides its consolidated usage as a host for protein production [18, 19]. Its advantageous traits include a low nutritional requirement, capacity to reach high cell densities, tolerance to acidic conditions, and the ability to utilize several carbon sources [20, 21]. Although *Komagataella* species are typically not known to assimilate xylose, recent findings demonstrated that certain strains can naturally metabolize xylose, albeit at slower rates [22]. This suggests a latent potential that can be harnessed through metabolic engineering. In the present study, *K. phaffii* was engineered to produce BTO from xylose. To achieve BTO production, the xylose oxidation pathway was introduced in the yeast along with the heterologous expression of a ketoacid decarboxylase. Then, a central composite design analysis (CCD) was designed to investigate the effect of dissolved oxygen levels and pH on enhancing BTO production in engineered *K. phaffii*. The effect of cell concentration was examined under the optimal conditions determined by the experimental design, providing an initial insight into the relationship between growth and BTO production.

## 2 Materials and Methods

### 2.1 Strains and Media

*E. coli* TOP10 (Invitrogen) was employed for cloning and amplification of plasmids. The bacteria strains were grown in low salt LB medium (1% tryptone, 0.5% NaCl, 0.5% yeast extract) containing the appropriate antibiotics (25 µg/mL zeocin, 100 µg/mL ampicillin, 50 µg/mL kanamycin, or 50 µg/mL hygromycin B). The plasmids and yeast strains used in this work are listed in Table 1. Yeasts were grown in YP medium (1% yeast extract, 2% peptone) supplemented with glucose (YPD) and/or xylose (YPDX or YPX), and the specific antibiotics when appropriate (100 µg/mL zeocin, 200 µg/mL geneticin, and 200 µg/mL hygromycin B). Alternatively, the FM22 mineral medium supplemented with different glucose and xylose concentrations was also employed in yeast cultivations [23]. When appropriated, the media were solidified with 2% agar. Cultivations were carried out at 37°C, 200 rpm or 30°C, 200 rpm, for *E. coli* or *K. phaffii* strains, respectively, unless later mentioned.

**TABLE 1:** *Komagataella phaffii* strains and plasmids used in this study.

Plasmid	Relevant characteristics		Reference
pGAPZB‐XylB‐HL	ZeoR, integrative plasmid carrying the *XylB‐HL* gene from *H. lutea*		[4]
pKGFP‐ld	KanR, integrative plasmid		[24]
pKLD	KanR, pKGFP‐ld derivative without *EGFP* gene (empty integrative plasmid)		[7]
pKLD‐XylD	KanR, pKLD derivative carrying XD‐*xylD* gene from *C. crescentus*		[7]
pKLD‐HL	KanR, pKLD derivative carrying XD‐*HL* gene from *H. lutea*		[7]
B3036	HygR, episomal plasmid		[25]
B3p	HygR, B3036 derivative obtained after XhoI/SacI digestion (empty episomal plasmid)		This work
B3p‐KDC	HygR, B3p derivative carrying *kivD* gene from *L. lactis*		This work
Strain reference	Relevant genotype	Relevant phenotype	Reference
X‐33	Wild type	Prototrophic	Invitrogen, USA
X‐33 HL	X‐33 + pGAPZB‐XylB‐HL	Expresses XDH, ZeoR	[4]
X‐33 HL pKLD	X‐33 + pGAPZB‐HL and pKLD	Expresses XDH, ZeoR and KanR	[7]
X‐33 HL XylD	X‐33 + pGAPZB‐HL and pKLD‐XylD	Expresses XDH and XD‐CC, ZeoR and KanR	[7]
X‐33 HL HL	X‐33 + pGAPZB‐HL and pKLD‐HL	Expresses XDH and XD‐HL, ZeoR and KanR	[7]
X‐33 HL pKLD KDC	X‐33 + pGAPZB‐HL, pKLD and B3p‐KDC	Expresses XDH and KDC, ZeoR, KanR and HygR	This work
X‐33 HL XylD KDC	X‐33 + pGAPZB‐HL, pKLD‐XylD and B3p‐KDC	Expresses XDH, XD‐CC and KDC, ZeoR, KanR and HygR	This work
X‐33 HL HL KDC	X‐33 + pGAPZB‐HL, pKLD‐HL, and B3p‐KDC	Expresses XDH, XD‐HL and KDC, ZeoR, KanR and HygR	This work

### 2.2 DNA Manipulation and Yeast Transformation

Table 1 shows all plasmids used in the study. Genes *xylB* from *Halomonas lutea* (XDH), *xylD* from *Caulobacter crescentus* (XD: *xylD‐CC*), *xylD‐HL* from *H. lutea* (XD: *xylD‐HL*), and *KivD* (KDC) from *Lactococcus lactis* were codon‐optimized and synthesized by GenOne LTDA/SA. Three *K. phaffii* strains (X‐33 HL pKLD, X‐33 HL XylD, and X‐33 HL HL, see Table 1) previously constructed by our research group [7], expressing a partial metabolic route (from xylose oxidation to the formation of the KDX intermediate) was used in the subsequent genetic modification with the *kivD* gene. The *kivD* gene encoding the 2‐keto acid decarboxylase enzyme was synthesized under the control of the TEF2 promoter and CYC1 terminator and then subcloned into the vector pB3Hyg using *Bam*HI restriction sites, resulting in the plasmid pB3‐KDC.

Yeast transformations were performed by electroporation according to recommendations in the *Pichia* Expression manual (Thermo Fisher Scientific). Cells were selected in YPD plates supplemented with 1 M sorbitol and 200 µg/mL hygromycin B. To verify the transformants after selection, colony PCR and yeast plasmid extraction followed by PCR were carried out with the primers PucOri‐F (5′‐GATCCGGCAAACAAACCACC‐3′) and Cyct‐R (5′‐CGTACACGCGTCTGTACAGA‐3′). These primers amplified the full KDC gene, annealing before its promoter sequence and within the terminator sequence. The presence of one fragment amplified, with a size length of 2634 bp, confirmed the positive clones.

### 2.3 Cultivation and Fermentation Conditions

Yeast strains were recovered from glycerol stocks at −80°C and streaked in solid YPD plates containing 100 µg/mL zeocin, 200 µg/mL geneticin, and 200 µg/mL hygromycin B. Pre‐cultures were grown from single colonies in 10 mL YPD in 50 mL conical tubes at 30°C and 200 rpm. After 48 h, the inoculum was transferred to 500 mL flasks containing 100 mL of YPDX for overnight growth. Cells were centrifuged at 5000 × *g* for 5 min, washed once, and used to initiate the fermentative experiments. Fermentation was carried out in 250‐mL Erlenmeyer flasks containing 50 mL of YPDX (10 g/L yeast extract, 20 g/L peptone, 10 g/L glucose and 10 g/L xylose) or mineral medium FM22 supplemented with PTM4 [23] and glucose and xylose at concentrations as specified later. Hygromycin B was added to the media used to cultivate *K. phaffii* when appropriate. Media pH was kept at 5.5, unless mentioned. The assays were performed at 30°C and 200 rpm, at least in two independent biological replicates. Samples were collected during the experiment to determine cell growth, sugar consumption and metabolite formation.

### 2.4 CCD Design to Optimize Cultivation Conditions and Improve BTO Production

The effect of dissolved oxygen levels (ranging from 2% to 20% saturation) and pH (ranging from 5 to 8.5) on BTO production was examined using a CCD (*α* = 1.41). The 11 experiments were randomly distributed into three rounds and three replicates at the central point (one in each round to verify and consider the variability of repeated inoculum preparation). All assays were performed using mineral medium FM22 supplemented with PTM4, 20 g/L glucose and 20 g/L xylose. The experiments were carried out in a stirred tank reactor (Minifors, Infors HT, Switzerland) with a working volume of 350 mL, at an initial OD_600nm_ of 5, a constant temperature of 28°C, and agitation of 200 rpm. With the set point value adjusted for each experimental condition, the pH of the cultures was controlled by adding 5 M KOH, and dissolved oxygen levels were managed by adjusting the airflow rate through a cascade control mode while maintaining a constant agitation speed.

The experimental data obtained from the CCD experiments were statistically analyzed using STATISTICA 12.0 software (Statsoft, USA). Second‐order models were developed for cultivation times of 72 and 96 h, with a significance level set at 90%. To validate the model predictions, fermentations were conducted in triplicate under the maximal BTO production condition indicated by the models. Model performance was further evaluated by calculating the root mean square error (RMSE) and the normalized RMSE (nRMSE) between experimental and predicted BTO values. The RMSE was determined as the square root of the mean squared differences between experimental and predicted values, representing the absolute prediction error. The nRMSE was calculated by dividing the RMSE by the range of observed values, expressing the average error as a percentage of the experimental data span.

### 2.5 Effect of Initial Cell Concentration

Considering the same medium and experimental parameters previously described, and the optimized combination of pH and dissolved oxygen levels, the influence of initial DO concentrations (2.5, 5.0, 10.0, and 20.0) on BTO production was estimated.

To calculate specific rates, a previously established relationship of g_DCW_/L = 0.354 × OD_600nm_ was utilized. The concentration of BTO and the OD were monitored over time. The BTO production rate (mg/h) and the specific BTO production rate (mg/g_DCW_.h) were calculated using measurements from two consecutive sampling points, with the rates set at the midpoint of this sampling interval.

### 2.6 Analytical Methods

Fermentation samples were centrifuged (10,000 × *g*, 10 min) and supernatants were analyzed by High‐Performance Liquid Chromatography (Waters AcQuity UPLC H‐Class RID) using an Aminex HPX‐87H (Bio‐rad) column. The analytical runs (10 µL, 24 min) were performed with 5 mM H_2_SO_4_ as mobile phase (0.6 mL/min flow rate, 45°C). Xylose, glucose, xylitol, glycerol, acetic acid, ethanol, and 1,2,4‐butanetriol concentrations were determined using calibration curves. Xylonic acid was quantified using a PDA detector and a reverse‐phase column HSS T3 (1.8 µm; 2.1 × 150 mm). The analytical run was carried out for 15 min, using a gradient elution consisting of solvent A (0.01 M KH_2_PO_4_, pH 2.0) and solvent B (Methanol) with a flow rate of 0.3 mL/min, at 30°C. In the presence of xylonic acid, xylose could not be accurately determined in the samples using the HPLC method. In such cases, xylose was estimated indirectly by subtracting the xylonic acid (quantified by UV) from the combined xylose and xylonic acid peaks detected by RID. Cell growth was determined by absorbance at OD_600nm_ using a spectrophotometer (SpectraMax M2, Molecular Devices) and correlated with cell‐dry weight as described previously [7].

## 3 Results and Discussion

### 3.1 Implementation of the 1,2,4‐Butanetriol Biosynthetic Pathway in *K. phaffii*

Previously, we demonstrated that the Dahms pathway was functionally expressed in *K. phaffii*, leading to ethylene glycol and glycolic acid production by the engineered strains [7]. Exploring the non‐phosphorylating xylose oxidation pathway, we wondered whether *K. phaffii* could produce other relevant compounds, such as 1,2,4‐butanetriol (BTO) from the 2‐keto‐3‐deoxy‐D‐xylonate (KDX) intermediate.

To implement the BTO pathway in the yeast, in addition to the xylose dehydrogenase (XDH: *xylD‐HL* from *H. lutea*) and xylonate dehydratase (XD: *xylD‐CC* from *C. crescentus* or *xylD‐HL* from *H. lutea*) genes already expressed, two more enzymes are necessary: a 2‐keto acid decarboxylase and an aldehyde reductase (Figure 1). In previous reports, different KDC enzymes have been screened in *E. coli* [15] and *S. cerevisiae* [12], of which the genes *kivD* and *kdcA* from *L. lactis* have demonstrated the best activities on KDX substrate. Considering that, the *kivD* gene was selected for the decarboxylase step in the BTO biosynthetic pathway.

**FIGURE 2:**
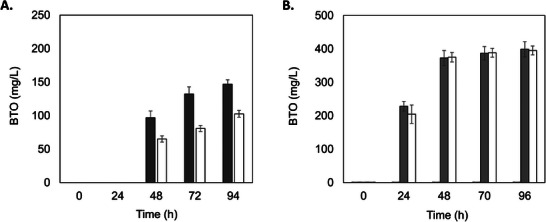
1,2,4‐butanetriol (BTO) production by the engineered *K. phaffii* strains. Cultivations of strains expressing the xylose dehydrogenase (*xylB‐HL*), xylonate dehydratase (*XylD‐HL* or *XylD‐CC*) and ketoacid decarboxylase (*kivD*) were carried out in shake flasks containing. (A) YP medium supplemented with 2 g/L glucose and 40 g/L xylose (white bars) or 10 g/L glucose and 10 g/L xylose (gray bars) with the strain X‐33 HL HL KDC; (B) mineral medium supplemented with PTM4, 10 g/L glucose and 10 g/L xylose with the strains X‐33 HL pKLD KDC (control, black bars), X‐33 HL XylD KDC (gray bars), X‐33 HL HL KDC (white bars). The experiments were kept for 94 (A) or 96 h (B), at 30°C and 200 rpm, with an initial cell density adjusted to OD_600nm_ ∼10. Results are means of biological duplicate experiments and error bars represent standard deviation (SD).

The expression of KDC was first evaluated in an X‐33 *K. phaffii* strain carrying the *xylB‐HL* (XDH) and *xylD‐HL* (XD) genes. During the cultivation of the recombinant strain, X‐33 HL HL KDC, in YPDX medium containing 2 g/L of glucose and 40 g/L of xylose or 10 g/L of both sugars, 100 to 150 mg/L of BTO were detected (Figure 2A). The production of BTO was slightly higher at lower sugar concentrations, while lower xylonic acid (∼5 g/L) was observed in this culture compared to the one with a high sugar concentration (∼10 g/L). No BTO production was detected in the control cells that do not express the dehydratase gene. The results demonstrate that *K. phaffii* possesses a native ALDR able to convert 3,4‐didydroxybutanal to BTO. Endogenous genes involved with the conversion of dihydroxybutanal into BTO have been identified in *E. coli* and *S. cerevisiae* [11, 15]. The overexpression of the aldehyde reductases yqhD (a NADPH‐dependent enzyme) or fucO and adhP (NADH‐dependent enzymes) in *E. coli* led to higher BTO production compared to the parental strain, suggesting a role of these native enzymes in the product formation [15]. Recently, by disrupting the *ADH* genes from *S. cerevisiae*, [11] identified Adh6 as the main enzyme responsible for catalyzing this reaction in the yeast. Alcohol dehydrogenases are promiscuous enzymes that catalyze reversible reactions of aldehydes to alcohols with low specificity [26]. Six alcohol dehydrogenases have been identified in *K. phaffii*, with *ADH900* and *ADH2* being the most transcribed genes [27]. These genes are responsible for ethanol production and methanol depletion in the yeast [26, 28]. However, no information is available on the substrate specificities of Adh(s) in *K. phaffii* other than the aforementioned substrates.

Since medium composition may contribute to the yeast performance and affect product yields, a mineral medium supplemented with PTM4 and 10 g/L of glucose and xylose, was also used to evaluate the BTO production by the yeasts. At this time, the effect of another xylonate dehydratase (XD) encoded by *xylD* from *C. crescentus* (*xylD‐CC)* was also investigated. As expected, the negative control strain X‐33 HL pKLD KDC, which does not have the XD expressed (incomplete pathway), could not produce BTO. In contrast, the strains carrying the complete pathway (XDH + XD + KDC), either with XD‐CC or XD‐HL produced the compound (Figure 2B). Under this condition, the strain carrying the XD encoded by *xylD‐HL* produced 395.4 mg/L of BTO, 2.7‐fold higher than the value obtained with the YP medium. A similar amount of BTO (398.9 mg/L) was observed in the recombinant yeast expressing the other *xylD‐CC* dehydratase‐encoding gene. As both dehydratase variants (*xylD‐HL* and *xylD‐CC*) led to comparable BTO titers under the tested conditions, X‐33 HL HL KDC was chosen for subsequent assays to further evaluate xylD‐HL, a novel xylonate dehydratase recently identified by our group [7].

**FIGURE 3:**
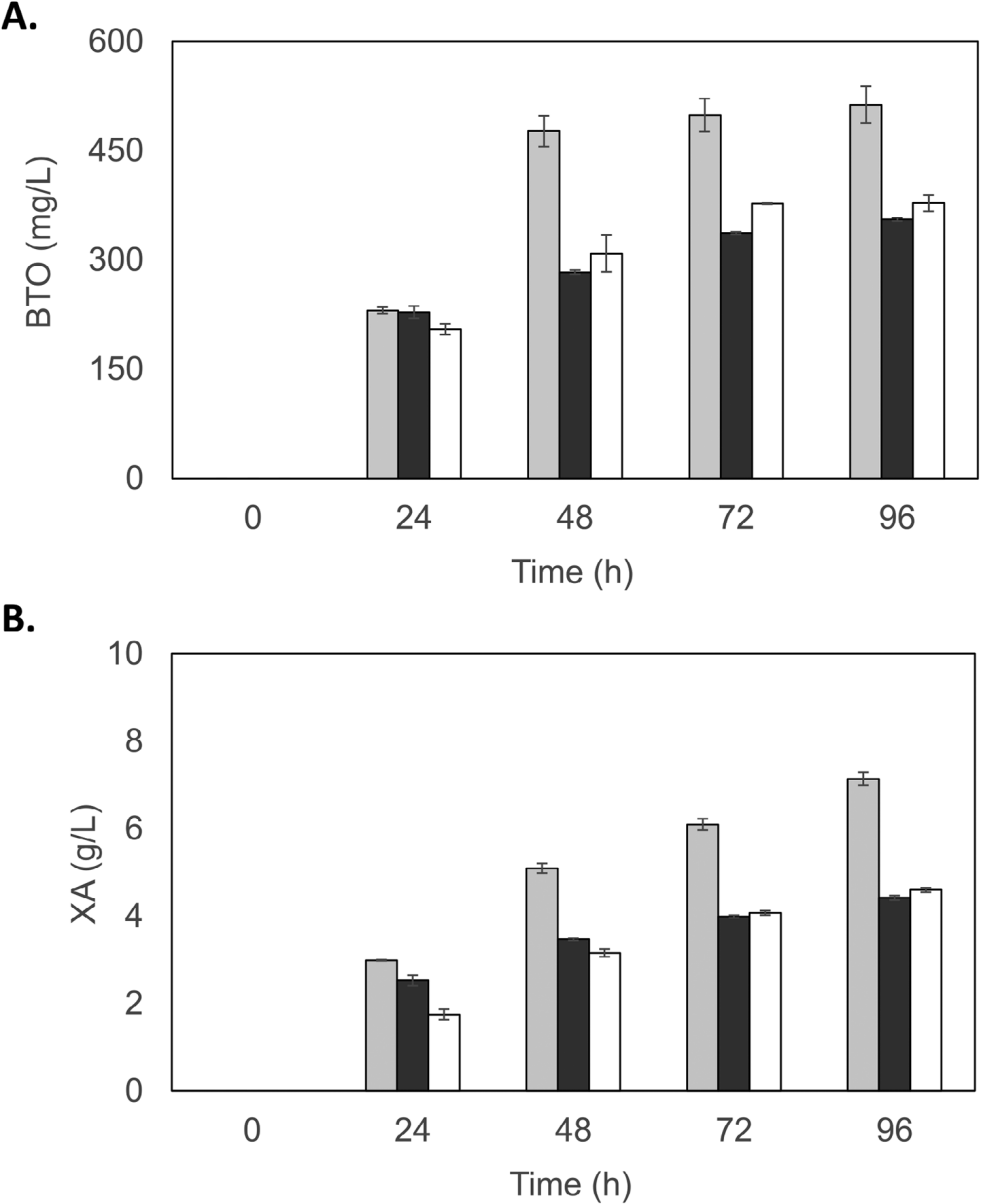
Effect of different sugar concentrations on the production of 1,2,4‐butanetriol (BTO) and xylonic acid (XA) by the engineered *K. phaffii* X‐33 HL HL KDC. Cultivations were carried out in shake flasks with mineral medium supplemented with PTM4 and glucose and xylose concentrations, respectively: 10/10 g/L (light gray bars), 10/20 g/L (dark gray bars) and 20/40 g/L (white bars). (A) BTO and (B) XA production. The experiments were kept for 96 h, at 30°C and 200 rpm, with an initial cell density adjusted to OD_600nm_ ∼10. Values represent averages ± standard deviation of two independent experiments.

To verify if sugar concentration affects product formation, the BTO‐producing strain was further compared in mineral media containing different glucose and xylose concentrations: 10/10 g/L, 10/20 g/L, and 20/40 g/L of glucose and xylose, respectively. For all conditions, glucose was completely consumed in the first 24 h (Figure S1). As shown in Figure 3, higher BTO production (513 ± 25 mg/mL) was observed when used 10 g/L of each sugar. At a higher xylose ratio, BTO formation dropped close to 70%. These results indicate that a higher glucose ratio favors BTO production. It is known that glucose consumption enhances glycolytic flux in engineered xylose‐consuming *S. cerevisiae* strains, indirectly boosting xylose consumption by improving the cell's energy and oxidative balance [29]. Xylonic acid accumulated in all tested conditions, indicating low XD activity, resulting in lower BTO yields. Indeed, the xylonate dehydration step has been reported as one of the main bottlenecks in the BTO biosynthesis pathway from xylose [12, 30]. Additionally, it has been observed that xylonic acid accumulation can adversely affect the performance of engineered microorganisms that use the non‐phosphorylation xylose oxidative pathway. This is because it leads to the acidification and toxicity of the media, which triggers a stress response in the strains. As a result, cell growth, enzymatic activities and product formation are affected negatively [31].

### 3.2 Optimization of BTO Production by *K. phaffii* Cells

Controlling pH acidification of culture media and oxygen supply improved 3,4‐DHBA in engineered *S. cerevisiae* cells using the xylose oxidative pathway [11]. In that study, lower agitation and, consequently, lower dissolved oxygen resulted in approximately 30% increase in product formation. The authors suggest that the improvement is related to XD activity since the dehydratase enzyme belongs to the IlvD/EDD protein family, which is iron‐sulfur oxygen‐sensitive. In this way, lower oxygen availability might maintain the XD functionality in the yeast cell, resulting in the increased product yields observed [11]. Considering that the enzyme XD has optimal activity around pH 8.0 [30], the study also tested different alkaline pHs for *S. cerevisiae* cultivation and found that a more alkaline pH resulted in re‐assimilation of xylonic acid, leading to an increase in product yield. Metabolome analysis of the implemented pathway showed a significant increase in KDX and dihydroxybutanal intracellular levels, probably due to higher XD activity in these conditions [11]. Previously, optimal pH for in vitro KDC activity was determined at pH 6.5, with significantly reduced activity at pH values over 7.5 and below pH 5.5 [32].

**FIGURE 4:**
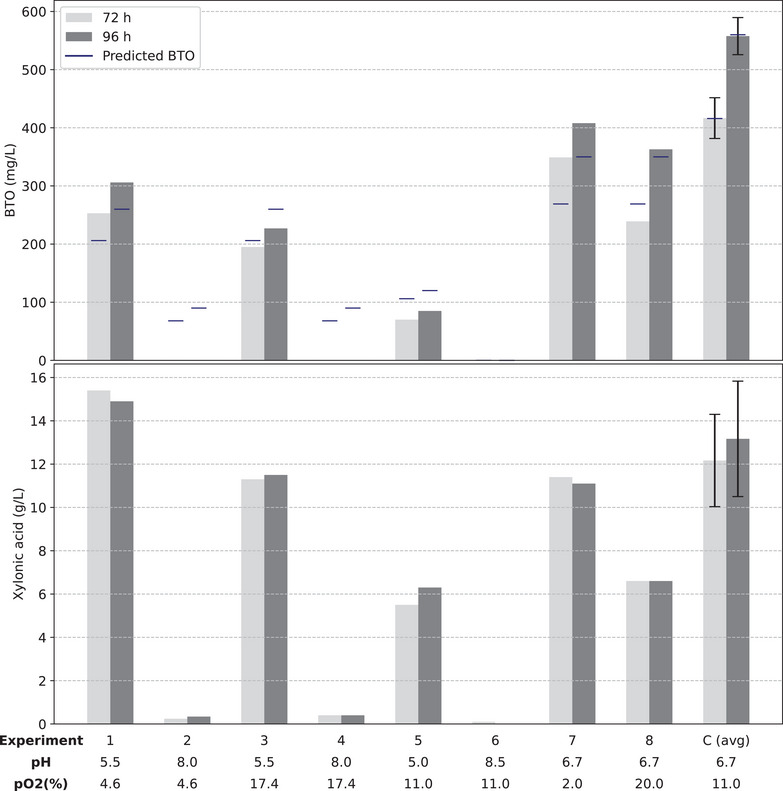
CCD experimental results for BTO production and xylonic acid accumulation in 72 and 96 h, as a function of pH and dissolved oxygen concentration (%). Central point replicates are presented as mean and standard deviation and identified as C (avg). Blue bars indicate the values predicted by the models for each experimental condition.

Therefore, a CCD design was used to determine the optimal combination of pH values (from 5 to 8.5) and dissolved oxygen concentrations (from 2% to 20%) to maximize BTO production by *K. phaffii* X‐33 HL HL KDC. Except for the variables under evaluation, all assays were conducted under uniform conditions, employing a mineral medium supplemented with PTM4, 20 g/L glucose, and 20 g/L xylose, with an initial OD_600nm_ of 5 at 28°C. Although 10 g/L of each sugar favored BTO production in shake flasks, concentrations were increased to 20 g/L in the bioreactor assays, maintaining the 1:1 sugar ratio, to prevent substrate depletion and support higher cell density during extended fermentations. The experimental conditions evaluated and the BTO concentration obtained for 72 and 96 h of fermentation are shown in Figure 4. The three replicates at the central point (presented as mean and standard deviation under the label C (avg) in Figure 4), distributed across the three rounds of experiments, resulted in BTO concentrations of 416 ± 42.9 mg/L after 72 h and 558 ± 38.8 mg/L after 96 h. The relative errors of 10% and 7%, respectively, indicate robust reproducibility.

The cultivations 2 and 4, performed at pH 8.0, demonstrated minimal cellular activity, with less than 5 g/L of glucose consumed and almost no xylose utilization over 96 h of cultivation (data not shown). Indeed, BTO was not produced at those conditions (Figure 4). Under condition 6, carried out at pH 8.5, no cellular activity was detected, as neither glucose nor xylose were consumed throughout the entire cultivation period. It is known that cultivating *K. phaffii* at a pH value above 8 significantly reduces cell viability [20].

A second‐order model was developed for each analyzed fermentation time using response surface methodology and experimental data. Equations (1) and (2) represent the regression equations for BTO production in 72 and 96 h, respectively, considering coded variables *X*_1_ (pH) and *X*_2_ (pO_2_), ranging from −1.41 to 1.41, and statistically significant parameters (*p* < 0.1).

(1) $$\begin{array}{ccc} \mathrm{BTO}\;72\;h\left(g/L\right) & = & 0.416-0.205.{\left(X_{1}\right)}^{2}-0.074.{\left(X_{2}\right)}^{2}\\ & & -\;0.069.\left(X_{1}\right),R^{2}=0.917 \end{array}$$

(2) $$\begin{array}{ccc} \mathrm{BTO}\;96\;h\left(g/L\right) & = & 0.558-0.278.{\left(X_{1}\right)}^{2}-0.106.{\left(X_{2}\right)}^{2}\\ & & -\;0.082.\left(X_{1}\right),R^{2}=0.924 \end{array}$$

The analysis of variance for the models revealed significant calculated *F* values for regressions. Specifically, the *F* ratios (*F*_calc_/*F*_tab_) were 4.6 and 6.5 for the 72‐ and 96‐h times, respectively. Additionally, the ANOVA indicated that the lack of fit was not significant, with F ratios of 0.25 and 0.15 for the respective times evaluated. The observed coefficients of determination (*R* ^2^) demonstrated a high level of agreement between experimental and simulated results. Based on this comprehensive analysis, we can confidently conclude that the models are statistically significant and suitable for predictive purposes. To further evaluate model performance, the root mean square error (RMSE) and normalized RMSE (nRMSE) were calculated using the predicted and experimental BTO values. The RMSE values were 53.72 mg/L for 72 h and 60.84 mg/L for 96 h, corresponding to normalized RMSEs of 11.55% and 10.17%, respectively. These results corroborate previous analyses regarding the predictive accuracy of the models and indicate that the average prediction errors account for a relatively small portion of the experimental response range, thereby supporting model applicability across the evaluated design space. The values predicted by each model for the respective fermentation times and experimental conditions are shown as blue lines in Figure 4.

**FIGURE 5:**
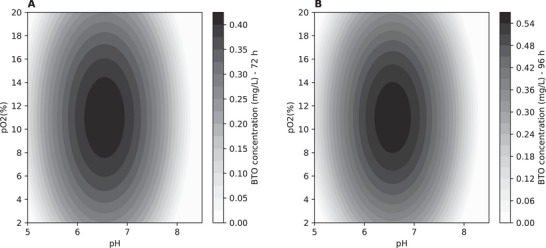
Contour plots for CCD response of BTO production at 72 h (A) and 96 h (B) as a function of pH and dissolved oxygen concentration.

Upon analyzing the equations described, it can be observed that the effect of dissolved oxygen concentration, within the evaluated range, is less significant in contributing to the responses compared to the effect of pH. Additionally, the models exhibited similarity in the magnitude and order of their effects. BTO concentration is maximized in both cases when maintaining dissolved oxygen concentration at 11% (*X*_2_ = 0). The pH value that maximizes the 72‐h response is pH = 6.54 (*X*_1_ = −0.168), while the maximization of the 96‐h response is achieved at pH = 6.57 (*X*_1_ = −0.147), which are similar values considering the imprecision of the pH control. The effect of pH and dissolved oxygen concentration on BTO production at 72 and 96 h of fermentation can be better observed in the contour plots presented in Figure 5.

To validate the maximum BTO production predicted by the model, an experiment was conducted at pH = 6.57 (*X*_1_ = −0.147) and pO_2_ = 11% (*X*_2_ = 0). Under these conditions, the models predicted BTO concentrations of 422 mg/L for 72 h and 564 mg/L for 96 h. Experimentally observed results, in triplicate, yielded concentrations of 407 ± 33 mg/L and 537 ± 24 mg/L for 72 and 96 h, respectively. The relative errors of 3.6% and 5.0% between observed and predicted values confirm the model's goodness of fit to the experimental data.

Unlike *S. cerevisiae*, *K. phaffii* could not re‐assimilate the xylonic acid exported from the cells. The optimum pH value for BTO production is sufficiently high to prevent the dissociation of the produced xylonic acid (pKa = 3.56) and its subsequent permeation through the membrane, thus mitigating the potential toxic effects on yeast. Transport of organic acids across the cell membrane is an important mechanism to maintain cellular homeostasis in the presence of acid compounds as well as allow the uptake of the acids to be used as nutrients by the cell [33, 34]. However, the mechanisms and genes specifically involved with xylonic acid transport remain unknown [5, 35]. In *S. cerevisiae*, TPO2 and TPO3 permeases play an important role in acetate efflux and tolerance to acetic acid [36] and JEN1, a monocarboxylate/proton symporter, mediates the uptake of several organic acids like lactate, pyruvate, and acetate [33]. Genes that respond to acetic acid were recently identified by analyzing the transcriptome of *K. phaffii* cells [37]. Up‐regulated genes include multidrug resistance (MDR) proteins, such as the polyamine transporter TPO1 and TPO3, and the monocarboxylate permease (GQ6701360) and other membrane transport proteins that are potentially involved in the efflux and/or uptake of several compounds in the yeast. Understanding the molecular mechanisms involved in xylonic acid export in *K. phaffii* could be crucial to further improve yields and titers in the engineered yeasts.

Ensuring adequate oxygen supply can indeed improve microbial conversion efficiency. Lower BTO production was detected when using pO2 levels of 2% (408 mg/L) or 20% (363 mg/L). Thus, a delicate balance of oxygen availability is required to provide the energy and cofactors NAD(P)H/NAD(P)^+^ necessary for the cells while maintaining the enzymatic activities of the metabolic pathway implemented at adequate levels.

### 3.3 Effect of Initial Cell Concentration

Since the CCD results suggest that the optimal condition would be close to pH 6.5 and pO_2_ 11%, the impact of different initial cell concentrations on BTO titers was then assessed under this condition. For this, the *K. phaffii* strain X‐33 HL HL KDC was cultivated in a mineral medium supplemented with PTM4, 20 g/L glucose and 20 g/L xylose, with initial yeast biomass OD_600_ varying from 2.5 to 20 under the previously described conditions.

**FIGURE 6:**
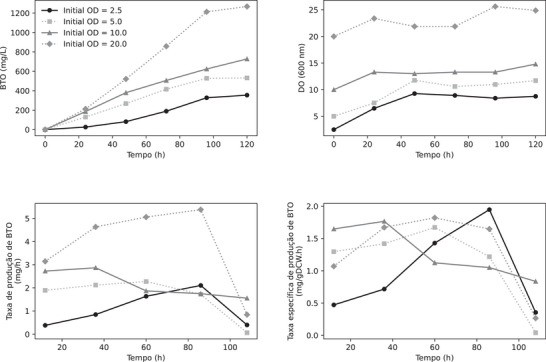
Effect of different initial cell concentrations on 1,2,4‐butanetriol (BTO) production by the engineered *K. phaffii* strain X‐33 HL HL KDC. BTO concentration (A), optical density (B), BTO production rate (C), and specific BTO production rate (D) as function of initial cell concentrations.

The experiment with an initial OD of 5.0, equivalent to the validation condition, was reevaluated. It resulted in BTO production of 416 mg/L in 72 h and 528 mg/L in 96 h. These figures correspond to relative errors of 1.5% and 6.8%, respectively, compared to the model‐predicted values, underscoring the robust reproducibility in preparing the inoculum, medium, and fermentation process. Figure 6 exhibits the profiles of BTO concentration (A), cell density (B), as well as the BTO production rate (C) and specific BTO production rate (D) obtained.

An almost linear BTO production profile was observed over time in all cases, with the maximum BTO production achieved with the highest initial cell density evaluated (OD_600_ = 20), reaching 1269 mg/L (Figure 6A). The optimization of two critical process parameters, pH and dissolved oxygen level, alongside an evaluation of cell concentration, yielded a substantial 147% enhancement in BTO production compared to the baseline condition initially observed (from 513 to 1269 mg/L). For initial ODs of 2.5 and 5, the 20 g/L of glucose initially available in the culture medium was completely consumed between 24 and 48 h of cultivation. For higher initial cell concentrations, glucose was completely consumed within the first 24 h of cultivation (data not shown). The availability of glucose is directly linked to cell growth, as OD increases while glucose is available to the cells (Figure 6B). Additionally, in all conditions, xylonate accumulation in the medium suggests that the sequential reactions converting xylonate to BTO may occur at reduced rates.

The maximum specific BTO production rate is observed at an average sampling time of 86 h for an OD of 2.5 (Figure 6C), whereas for higher cell concentrations, the maximum specific rate occurs in a shorter time frame (approximately 40 h) (Figure 6D). This suggests that optimal rates of BTO production can be achieved by maintaining a low glucose concentration during cultivation. Therefore, combining high OD values with a feeding strategy to keep low and constant glucose concentrations in the bioreactor could result in enhanced BTO titers and higher productivity.

## 4 Conclusion

The results presented in this work indicate strategies to enhance the production of BTO in *K. phaffii*. The pH and oxygen availability should be adjusted according to the specific pathway introduced and the host microorganism used. A neutral pH seems to be more appropriate for the functionality of the enzymes involved in the BTO pathway. Previously, BTO production has been evaluated in different bacteria and yeast strains (Table 2). The highest BTO titer (6.6 g/L) was achieved by a *S. cerevisiae* extensively engineered under fed‐bath fermentation. Although the BTO titer obtained by *K. phaffii* is lower (1.3 g/L), our work represents the first report of BTO production using this yeast. Given its industrial relevance, *K. phaffii* represents a promising platform for further metabolic engineering to improve BTO biosynthesis.

**TABLE 2:** Brief review of 1,2,4‐butanetriol (BTO) production from xylose by engineered microorganisms.

Strain	BTO (g/L)	Molar yield (%)	Experimental condition	Genetic modifications	Ref.
*E. coli* BL21	3.92	27.7	Fed batch fermentation. Mineral medium with 20 g/L xylose. Glycerol was used as co‐substrate	Overexpression of *xylB*, *xylC* from *C. crescentus*, *mdlC* from *P. putida, yjhG* and *adhP* from *E. coli*. Deletion of native genes *xylAB*, *yjhH* and *yagE*	[13]
*E. coli* WL3110	0.88	12	Shake flask fermentation. Mineral medium with 10 g/L xylose	Overexpression of *xylB* from *C. crescentus*, *mdlC* from *P. putida* and *adhP* from *E. coli*. Deletion of native genes *xylAB*, *yjhH* and *yagE*	(Valdehuesa et al., 2014)
*E. coli* BL21‐14	5.1	18	Shake flask fermentation. LB medium with 40 g/L xylose	Overexpression of *xylB*, *xylD*, *kdcA*, and *adhP*	[15]
*S. cerevisiae* BDδD2tkdcA	1.7	24.5	Batch fermentation. YP medium with 10 g/L glucose and 10 g/L xylose	Overexpression of *xylB* and *xylD* from *C. crescentus*, *kdcA* from *Lactococcus lactis*, and truncated *TYW1*. *xylD* and *kdcA* were integrated in multiple copies. Deletion of native *GRE3*	[12]
*S. cerevisiae* BDδK6035	6.6	57	Fed‐batch fermentation. YP medium with glucose and xylose	Overexpression of *xylB* and *xylD* from *C. crescentus*, *kdcA* from *Lactococcus lactis*, native *ADH6* and truncated *TYW1 and POS5*Δ17 NADH kinase. *xylD* and *kdcA* were integrated in multiple copies. Deletion of native *GRE3* and *BOL2*	[17]
*C. tropicalis* BT19	4.0	—	Batch fermentation. YP medium with 30 g/L glucose and 30 g/L xylose, supplemented with 1 mm ammonium ferric citrate and 9 g/L calcium carbonate	Overexpression of *xylB, xylD*, and *kdcA*, with modulation of iron metabolism and NADPH regeneration pathway. Deletion of native *GRE3*, *XYL2* and *XKS* genes	[16]
*K. phaffii*	1.3	—	Batch fermentation. Mineral medium with 20 g/L glucose and 20 g/L xylose	Overexpression of *xylB* and *xylD‐HL* from *H. lutea* and *kivD* from *L. lactis*	This work

This study presents the implementation of a synthetic metabolic pathway for BTO production from xylose in the yeast *K. phaffii*. The data show oxygen availability, pH, and initial cell density's contribution to product formation. The strain yielded 1.3 g/L of BTO using a mineral medium comprising an equimolar mixture of glucose and xylose as carbon sources, under optimized conditions defined by a CCD design (pH 6.57 and pO_2_ of 11%). The systematic optimization of these critical process parameters, complemented by a cell density evaluation, led to a remarkable 147% enhancement in BTO production compared to the condition initially observed. Further investigations are necessary to improve the yields and titers of BTO production in this host yeast. Multicopy integration of XD and KDC encoding genes, overexpression of native ADH or aldehyde reductases involved in the last step, attenuation of competing genes, or even deletion or expression of xylonate permeases, are strategies that might be explored to improve the biotechnological process.

## Author Contributions

**Débora Trichez and Thályta Pacheco**: conceptualization, investigation, data analysis, writing – original draft, review and editing. **Clara Vida G. C. Carneiro**: investigation, data analysis. **Jessica C. Bergmann**: conceptualization, investigation. **João Ricardo M. de Almeida**: conceptualization, supervision, writing – review and editing, funding acquisition. All authors reviewed the manuscript.

## Conflicts of Interest

The authors declare no conflicts of interest.

## Supporting information




**Supporting file 1**: biot70085‐sup‐0001‐SuppMat.docx

## Data Availability

The data that support the findings of this study are available on request from the corresponding author. The data are not publicly available due to privacy or ethical restrictions.
