# Supplementary material for: Metabolic Engineering of Komagataella phaffii and Process Optimization for Biosynthesis of 1,2,4‐Butanetriol From Xylose
Source: Biotechnol J. 2025 Jul 25;20(7):e70085. doi: 10.1002/biot.70085 (PMC12292740; doi:10.1002/biot.70085)

**Supplementary data**

**Metabolic Engineering of *Komagataella phaffii* and Process Optimization for Biosynthesis of 1,2,4-Butanetriol from Xylose**

Débora Trichez¹, Thályta Pacheco¹^,^²; Clara Vida G. C. Carneiro¹^,^², Jessica C. Bergmann¹, and João Ricardo M. de Almeida¹^,^²*

^1^ Microbial Genetics and Biotechnology Laboratory, EMBRAPA Agroenergy, Brasília, Brazil

^2^ Graduate Program of Microbial Biology, Institute of Biology, University of Brasília, Brazil

***Corresponding author**

João Ricardo Moreira de Almeida

EMBRAPA Agroenergia

Parque Estação Biológica, PqEB – W3 Norte Final s/n^o^

70.770-901 – Brasília, DF – Brasil.

**E-mail:** joao.almeida@embrapa.br

**Tel:** +55 61 3448-2337

**Figure S1.** Effect of different sugar concentrations on the production of 1,2,4-butanetriol (BTO) by the engineered *K. phaffii* X-33 HL XylD-HL KDC. Cultivations were carried out in shake flasks with mineral medium supplemented with PTM4 and glucose and xylose concentrations, respectively: (A) 10/10 g/L, (B) 10/20 g/L, and (C) 20/40 g/L. The experiments were kept for 96h, at 30ºC and 200 rpm, with an initial cell density adjusted to OD600nm ~10. Values represent averages ± standard deviation of two independent experiments.


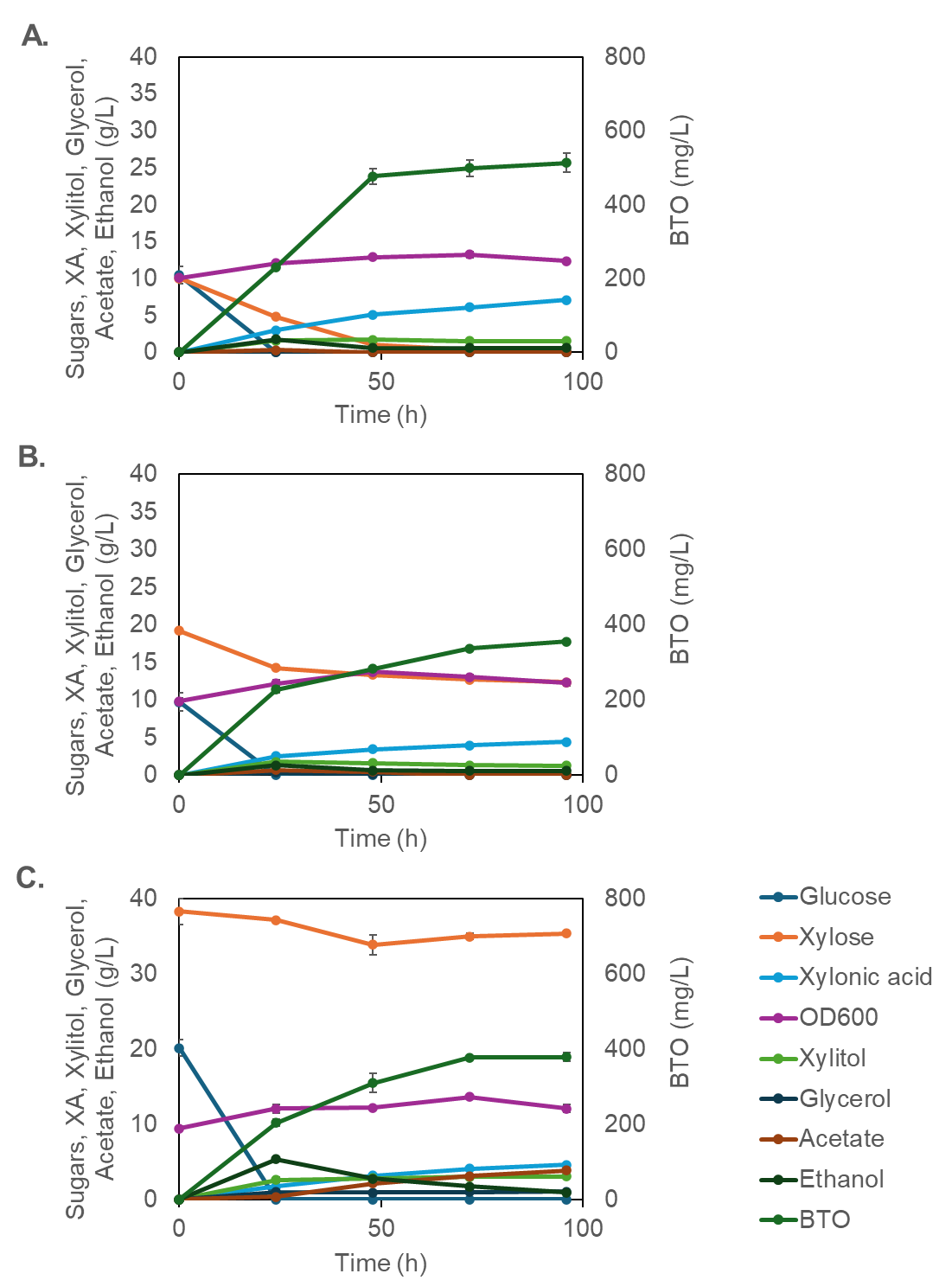

Supplement: Supplementary file 1 — Supporting file 1: biot70085‐sup‐0001‐SuppMat.docx [file BIOT-20-e70085-s001.docx]
